# Median arcuate ligament syndrome diagnosis on Computed Tomography: what a radiologist needs to know

**DOI:** 10.1016/j.radcr.2021.06.093

**Published:** 2021-09-16

**Authors:** Pooja Narwani, Navin Khanna, Ishwariya Rajendran, Hesham Kaawan, Rafid Al-Sam

**Affiliations:** a Department of Radiology, The Pennine Acute Hospitals NHS Trust, UK; b Department of Medicine, The Pennine Acute Hospitals NHS Trust, UK

**Keywords:** Median arcuate ligament syndrome (MALS), Celiac artery compression, Vascular compression syndrome, Diagnostic Radiology, Computed Tomography (CT), Ultrasound Doppler, Celiac artery stenosis

## Abstract

Median arcuate ligament syndrome or celiac artery compression syndrome is one of the abdominal vascular compression syndromes due to compression of proximal celiac artery by the median arcuate ligament. The median arcuate ligament unites diaphragmatic crura on either side at the level of aortic hiatus. The ligament has a low insertion causing compression of the celiac artery resulting in clinical symptoms of postprandial pain and weight loss. It is a rare syndrome, detected incidentally on routine Computed Tomography abdomen and pelvis studies. We present a rare case of a 35-year-old female who presented with abdominal pain. She was evaluated by Computed Tomography scan of the abdomen and pelvis. Ultrasound Doppler of mesenteric vasculature helped detect celiac artery stenosis. A referral to the vascular surgery department was made; however, the patient was managed conservatively.

The median arcuate ligament is a fibrous arch that unites diaphragmatic crura on either side of the diaphragm at the level of aortic hiatus. Usually, the ligament passes superior to the celiac artery, causing no compression. The ligament can have a low insertion, resulting in compression of the proximal celiac artery, causing postprandial abdominal pain and bruit symptoms. This article discusses the characteristic findings of distinct narrowing of proximal celiac artery and post stenotic dilatation caused by low lying median arcuate ligament on Computed Tomography. Ultrasound Doppler of the abdomen can help detect celiac artery stenosis.

## Case report

**Fig. 1:**
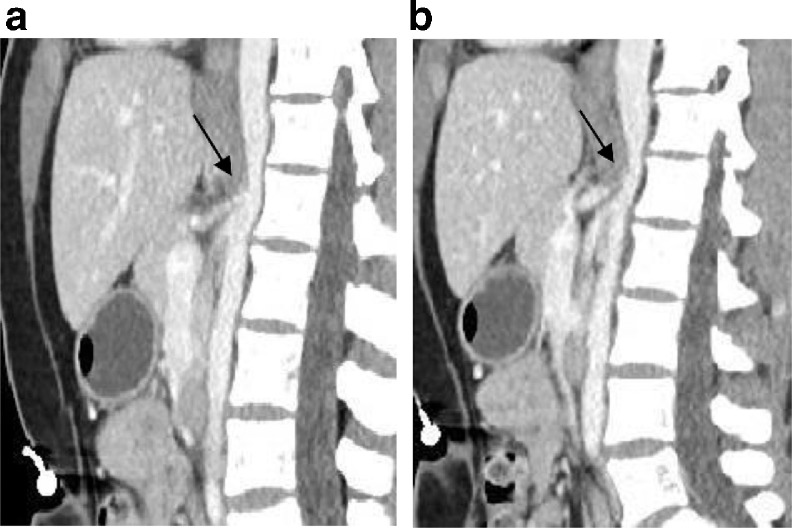
(A): Contrast-enhanced CT of the abdomen, parasagittal reconstruction, shows kinking of the proximal celiac artery, resulting in characteristic hooked appearance with post stenotic dilatation. Note the absence of atherosclerosis. (B): Contrast-enhanced CT of the abdomen, parasagittal reconstruction, shows post stenotic dilatation of the celiac artery.

**Fig. 2:**
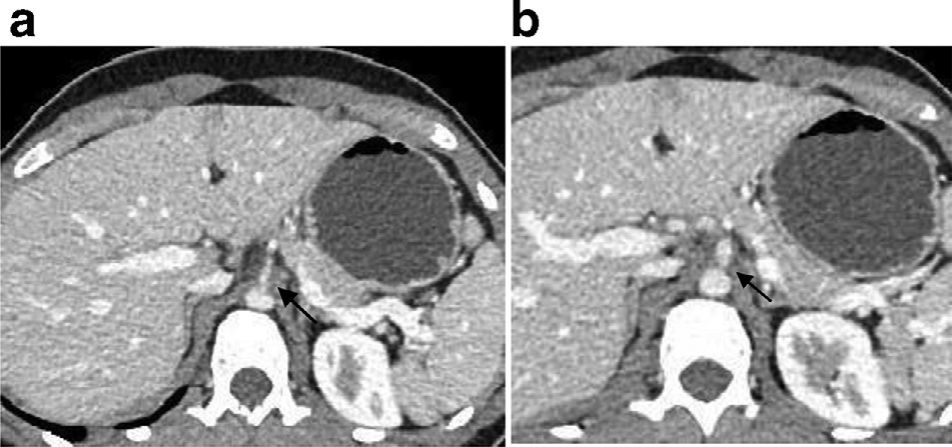
(A): Contrast-enhanced CT axial image of the abdomen shows proximal stenosis of the celiac artery. (B): Contrast-enhanced CT axial image of the abdomen shows proximal stenosis accompanied by post stenotic dilatation, although it is better appreciated on Sagittal CT image.

A 35-year-old lady presented in emergency department with symptoms of abdominal pain and weight loss. On physical examination, she had mild tenderness in the epigastric region, and her laboratory studies were within normal limits. She had no co-morbidities and was evaluated by a routine Computed Tomography (CT) scan of the abdomen and pelvis. A single venous phase CT scan was acquired 60 sec after contrast injection. Images were reconstructed in all three planes. CT images, particularly sagittal images, revealed narrowing of proximal celiac artery by obliquely oriented soft tissue band anterior to the abdominal aorta at L1 level. The median arcuate ligament could be identified uniting with the diaphragmatic crura on both sides (Figs. 1A and B). There was associated post stenotic dilatation with proximal pre stenotic segment (Figs. 2A and B). The superior and inferior mesenteric arteries were unremarkable with no stenosis.

**Fig. 3:**
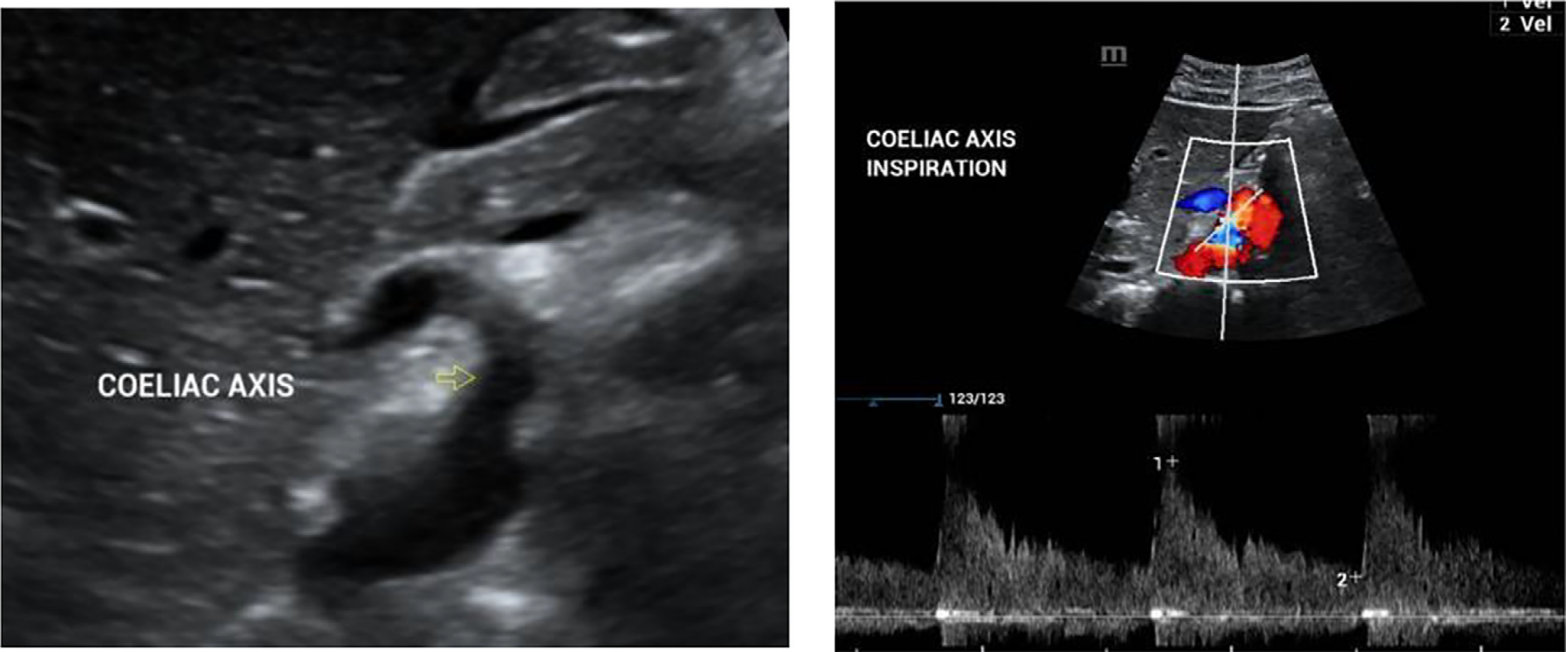
(A): Ultrasound Abdomen image shows the origin of celiac artery from the aorta. (B): Ultrasound Color Doppler with spectral analysis of coeliac artery shows elevated PSV >250 cm/s during inspiration. Note the turbulent flow in the post stenotic segment.

Later, she was referred to a vascular surgeon, who advised Ultrasound Doppler of the abdomen to confirm the diagnosis. Ultrasound Doppler showed high velocities in the celiac artery, more than 250 cm/s on average. Due to its dynamic nature, ultrasound was performed in inspiration and expiration, showing high velocities in both phases of respiration (Figs. 3A and B). The patient was diagnosed with median arcuate ligament syndrome after careful correlation with clinical symptoms, physical examination and imaging findings. She was, however, managed conservatively.

## Discussion

Median arcuate ligament syndrome (MALS) is one of the abdominal vascular compression syndromes diagnosed by Harjola in 1963 [1]. It is more commonly known as celiac artery compression syndrome, also known as Dunbar syndrome, named after the radiologist JD Dunbar [2]. It typically affects young women in the ratio of 2:1 to 3:1 and age group of 20–40 years of age with a reported incidence of 2 per 100,000 patients [3]. Usually, the median arcuate ligament crosses at L1 above the origin of the celiac artery. In 10%–24% of the general population, MAL can have a low insertion; however, even a smaller subset will develop symptoms due to celiac artery compression [4]. Typical physical examination findings include abdominal bruit in a mid-epigastric region that varies with respiration.

Pathophysiology: In MALS, the pathophysiology is vascular due to compression of the celiac artery causing foregut ischemia, vascular steal phenomenon causing midgut ischemia with splanchnic vasoconstriction and ischemia. Some authors think the aetiology is neurogenic due to the compression of celiac plexus and ganglion (5).

## Radiological findings

Modern *Multidetector **Computed Tomography*** **(CT)** scanners offer several advantages such as easy availability, non-invasive nature, multiplanar reconstruction, superior resolution and faster image acquisition, thereby; making it a preferred imaging modality. Classical findings include narrowing in the proximal celiac artery with inferior displacement due to indentation by median arcuate ligament resulting in characteristic hooked configuration. It is best appreciated on sagittal images and can be easily overlooked on the axial images. The typical hooked configuration helps to differentiate from atherosclerosis [6]. Secondary indirect signs include post stenotic dilatation and vascular collateral channels from SMA. Median arcuate ligament thickness of more than 4 mm is abnormal.

Also, note that CT studies are best evaluated in the end-inspiratory phase. Since MAL is attached to the diaphragm, movement occurs with respiration, and true compression can be evaluated in the end-inspiratory phase. Isolated compression of the celiac axis in expiration can be observed in 13%–50% of healthy individuals and can be clinically insignificant. Few of these patients would have clinical symptoms due to hemodynamic compromise [6,7]. In a retrospective study, Heo et al. showed that 87% of patients with classical imaging findings of MALS incidentally detected on CT had no symptoms [8].

***Doppler ultrasound*** with peak systolic velocity of 200 cm/s can have a reported sensitivity and specificity of 75% and 89%, respectively [9]. Ultrasound Doppler, due to its dynamic nature, is performed in different phases of respiration.

***Conventional angiography*** Findings are similar to CT with superior indentation of celiac artery, typical hooked configuration and post stenotic dilatation. In some cases, retrograde filling of the celiac artery from collaterals arising from SMA noted. Conventional Angiography was used in the past to make a diagnosis of this syndrome.

## Management

Cienfuegos et al. [10] suggested the criteria for laparoscopic resection of the ligament in young patients, with symptoms of severe postprandial pain, >70 % stenosis of celiac artery and development of collateral circulation. A smaller subset may need vascular reconstruction due to vascular damage caused by compression of the celiac artery [11].

## Conclusion

Low lying median arcuate ligament is an important anatomical variant, and one should exercise caution in making the diagnosis unless backed by supporting clinical features. Diagnosis is based on characteristic CT findings of proximal celiac artery narrowing with hooked shaped configuration by the median arcuate ligament. Management includes laparoscopic resection of the median arcuate ligament.

## Learning points

-Median arcuate ligament syndrome should be diagnosed carefully in combination with clinical and imaging findings. Low insertion of the median arcuate ligament is an important anatomical variant and can be seen in asymptomatic individuals.

-Classical imaging findings include compression of proximal celiac artery by the ligament with associated post stenotic dilatation resulting in the characteristic hooked shaped configuration.

-MALS can be easily missed on routine CT, so the radiologist should carefully assess the multiplanar images to establish the diagnosis.

## Patient consent

A written informed consent for the publication of this case has been taken from the patient. A consent form can be attached if required.

## References

[1] Harjola PT. (1963). A rare obstruction of the coeliac artery. Ann Chir Gynaecol Fenn.

[2] Dunbar JD, Molnar W, Beman FF, Marable SA (1965). Compression of the celiac trunk and abdominal angina. Am J Roentgenol Radium Ther Nucl Med.

[3] Duran M, Simon F, Ertas N, Schelzig H, Floros N. (2017). Open vascular treatment of median arcuate ligament syndrome. BMC Surg.

[4] Linder HH, Kemprud E. (1971). A clinicoanatomic study of the arcuate ligament of the diaphragm. Arch Surg.

[5] Weber JM, Boules M, Fong K, Abraham B, Bena J, El-Hayek K (2016). Median arcuate ligament syndrome is not a vascular disease. Ann Vasc Surg.

[6] Horton K.M., Talamini M.A., Fishman E.K. (2005). Median arcuate ligament syndrome: evaluation with CT angiography. Radiographics.

[7] Patten R M, Coldwell D M, Ben-Menachem Y (1991). Ligamentous compression of the celiac axis: CT findings in five patients. Am J Roentgenol.

[8] Heo S, Kim HJ, Kim B, Lee JH, Kim J, Kim JK. (2018). Clinical impact of collateral circulation in patients with median arcuate ligament syndrome. Diagn Interv Radiol.

[9] Ozel A,Toksoy G, Ozdogan O, et al.Ultrasonographic diagnosis of median arcuate ligament syndrome: a report of two cases. Med Ultrason 2012; 14:154.7. 22675717

[10] Cienfuegos JA, Estevez MG, Ruiz-Canela M (2018). Laparoscopic treatment of median arcuate ligament syndrome: analysis of longterm outcomes and predictive factors. J Gastrointest Surg.

[11] Jimenez JC, Harlander-Locke M, Dutson EP. (2012). Open and laparoscopic treatment of median arcuate ligament syndrome. J Vasc Surg.

